# The effectiveness of using entertainment education narratives to promote safer sexual behaviors of youth: A meta-analysis, 1985-2017

**DOI:** 10.1371/journal.pone.0209969

**Published:** 2019-02-12

**Authors:** Victor Orozco-Olvera, Fuyuan Shen, Lucie Cluver

**Affiliations:** 1 Development Research Group, The World Bank, Washington DC, United States of America; 2 Bellisario College of Communications, The Pennsylvania State University, State College, Pennsylvania, United States of America; 3 Department of Social Policy and Intervention, University of Oxford, Oxford, United Kingdom; Cardiff University, UNITED KINGDOM

## Abstract

**Background:**

Risky sexual behaviors are associated with the transmission of sexually transmitted infections (STIs) and unwanted pregnancies, both major health concerns for youth worldwide. This review studies the effectiveness of narrated mass media programs in promoting safer sexual practices among youth in developed and developing countries.

**Methods:**

Electronic and manual searches were conducted to identify experimental and quasi-experimental studies with robust counterfactual designs published between 1985 and the first quarter of 2017. Effect sizes were meta-analyzed using mixed-effects models.

**Results:**

Eight experimental and two quasi-experimental studies met our inclusion criteria. The aggregated sample size was 23,476 participants, with a median of 902 participants per study. Entertainment education narratives had small but significant effects for three sexual behaviors. It reduced the number of sexual partners [standardized mean difference, (SMD) = 0.17, 95% confidence interval (CI) = 0.02–0.33, three effect sizes], reduced unprotected sex (SMD = 0.08, 95% CI = 0.03–0.12, nine effect sizes), and increased testing and management for STIs (SMD = 0.29, 95% CI = 0.11–0.46, two effect sizes). The interventions were not effective in reducing inter-generational sex, measured through the age-gap with sexual partners (SMD = 0.06, 95% CI = -0.06–0.19, four effect sizes). Entertainment education had medium-size effects on knowledge outcomes (SMD = 0.67, 95% CI = 0.32–1.02, seven effect sizes), where a time-decay relationship is observed. No effects were found on attitudes.

**Conclusion:**

Although mass media entertainment had small effects in promoting safer sexual practices, its economies of scales over face-to-face interventions suggest its potential to be a cost-effective tool above an audience threshold. The use of study participants from the general youth population and the use of mostly effectiveness trials mitigate concerns regarding its scalability. The overall paucity of high-quality studies affirms the need for strengthening the evidence base of entertainment education. Future research should be undertaken to understand the moderator effects for different subgroups and intervention characteristics.

## Introduction

Risky sexual behaviors are associated with the transmission of STIs and unwanted pregnancies, both major health concerns for youth worldwide[1]. Sexual intercourse remains the main transmission mechanism of HIV among youth [2, 3], with inter-generational sex between young women and older men being an important driver in low-income contexts [4,5]. Teenage pregnancies are associated with reduction in socio-economic wellbeing of the mother and her children [5,6,7,8,9]. Systematic reviews of HIV prevention interventions suggest that while these are effective in developed countries, they are usually not in developing countries [10,11,12,13,14,15]. With HIV prevention funds declining in the last decade [16], it is important to invest in effective prevention programs.

Government and development partners have used mass media to persuade youth to engage in safer sexual practices [17,18]. Mass media entertainment in a narrative format (e.g., dramas, sitcoms), in contrast to informational programs delivered by media (e.g., television public service announcements) is a type of entertainment education. Though the latter is traditionally defined as the purposeful use of mass media to promote development objectives [18,19], entertainment education also includes commercial productions with prosocial messages that lack social objectives. Social learning theory [20], the main psychological theory underpinning entertainment education narratives, argues that show characters may be potential role models; and that they may help improve audiences’ self-efficacy for adopting new behaviors [21]. Other theories emphasize its potential to reduce counter-arguing when content may be counter-attitudinal [22,23] and updating perceptions of prevalent social norms [24,25]. Entertainment education has received increased attention in international development given the limited effectiveness of traditional behavior change programs across different fields, including HIV [26].

It is a longstanding debate on how exposure to entertainment media may affect sexual behaviors [27,28]. While observational evidence suggests a relation [29,30,31], determining whether such exposure influences beliefs and behaviors with observational data is empirically difficult due to sample-selection issues [32, 33]. The sexual beliefs and behaviors of a program’s audience may differ from those of individuals who decide not to watch the show; preventing studies to measure credible impact estimates. In the last decade, a series of evaluations have emerged that better address selection issues [34,35, 36]. Although sexual health is among the most common applications of entertainment education [37,38], the evidence base remains mostly qualitative [39]. Previous systematic reviews concluded that mass media interventions had small to medium effects on HIV testing [40] and risky sexual behaviors [41,42,43]. These reviews highlight the lack of high-quality evidence in this field.

This meta-analysis aims to assess the effectiveness of entertainment education narratives on promoting HIV testing and reducing risky sexual behaviors of youth in developed and developing countries. Youth was defined as individuals between 15 and 24 years of age. The scope of this review is narrower with respect to previous reviews of mass media interventions with sexual health objectives. This review will (i) be restricted to the mentioned age group, as its psychological development, relationship characteristics and sexual behaviors is likely to differ from those of older age cohorts (e.g., single individuals are likely to have a greater number of sexual partners, where condom use is more relevant); (ii) include experimental and quasi-experimental studies that have strong identification strategies following Cochrane Review guidelines (i.e., we exclude studies where selection bias is likely to persist); (iii) interventions that have a narrative format (e.g., we exclude for example flyers and public service announcements that lack this format); (iv) preferably include effectiveness trials were the type of program exposure follows that of the scaled-up intervention. For example, “voluntary” take-up for community screenings or “imposed” exposure for school-based screenings; and (v) studies that measured actual rather than intended behavior change by excluding studies that collected post-intervention data immediately after program exposure.

## Methods

This review follows reporting standards for the Preferred Reporting Items for Systematic Reviews and Meta-Analyses (PRISMA) [44]. The PRISMA checklist is in S1 Table. This review was registered in the PROSPERO International prospective register of systematic reviews (PROSPERO 2016: CRD42016046005).

### Selection criteria

Following Cochrane Review standards, studies were eligible for inclusion if they were randomized controlled trials (RCT), cluster RCTs, and quasi-experimental studies with robust counterfactual designs, including interrupted time series designs with control groups. We included studies that measured outcomes at least one week after exposure. Studies of complex interventions (those that included other information components, such as HIV information) were included if the edutainment component played a prominent part. By homogenizing the interventions targeting youth, the review aims to better identify the effectiveness of the narrated or storytelling format.

### Search strategy

Published studies were searched in English. The search period covered 1985 to the latest available year. We completed our searches on March 31, 2017. The following databases were searched: Communication & Mass Media Complete, EBSCO, ERIC (CSA), EconLit, JSTOR, MEDLINE, CINAHL (EBSCO), JSTOR, metaRegister of controlled trials, NBER, OpenSIGLE (grey literature), PsycINFO, RePEC, and The Cochrane libraries. The full search strategy of the MEDLINE database is provided as S1 List. References from previous reviews and from retrieved articles were examined for additional studies. Journal articles, published reports and for the case of economics research, published working papers were included (prior to journal publication, the latter are standard practice in the economics literature). Authors were contacted for further information when required.

### Risk of bias assessment

Study quality was assessed using the Cochrane Review guidelines for assessing risk of bias [45]. Each study was evaluated for participant selection (sequence generation and allocation concealment), blinding (personnel and outcome assessors), attrition (≥ 80% retention), and selective reporting and other types of biases. Each item was scored as either low, high or unclear risk of bias.

### Data analysis

For cluster RCTs or natural experiments delivered that failed to cluster their standard errors; we used a conservative value for the intra-class correlation coefficients of 0.05 [46]. We preferred intent-to-treat rather than per protocol estimates to minimize potential selection issues.

Standard meta-analytical methods were used. We used the Comprehensive Meta-Analysis software to convert all binary and continuous data outcomes to standardized mean differences and their 95% confidence intervals [47,48]. The data was coded so that safer sexual behaviors translated into positive values. For studies that reported different types of primary behaviors, outcome values were aggregated into four behavior categories: (i) age-gap with sexual partners; (ii) number of sexual partners (iii) unprotected sex; and (iv) STI testing and follow up.

The primary analysis for each of the four behaviors includes some degree of heterogeneity as it synthesizes data from non-identical interventions and from similar but different outcome indicators. Study heterogeneity is recognized by applying the mixed-effects model [49] and by calculating the I^2^ statistics [45, 50]. Our mixed-effects models report statistics based on random-effects weights within and not between subgroups. The meta-analysis used the study as the unit of analysis and the mean of the selected outcomes (we did not assume independence of outcomes within each study). Each included study only had one-time point. The I^2^ statistic describes the percentage of the variability in effect estimates that is due to heterogeneity rather than sampling error.

To determine whether results were robust to methodological decisions, we re-ran the analysis for the primary outcomes by repeating the meta-analysis using fix-effects models; we removed one study at a time to determine if it substantially affected the meta-analyzed effect size, and we re-ran the analysis without studies that were rated with high risk-of bias (defined as having more than two out of seven items evaluated at high-risk). To further assess the likely non-independence of outcomes within a study, we ran a random-effects model combining all behavior outcomes. We assessed whether publication bias was present through funnel plots [51]and Rosenthal’s [52] and Egger’s tests [53].

## Results

### Results of the search

**Fig 1:**
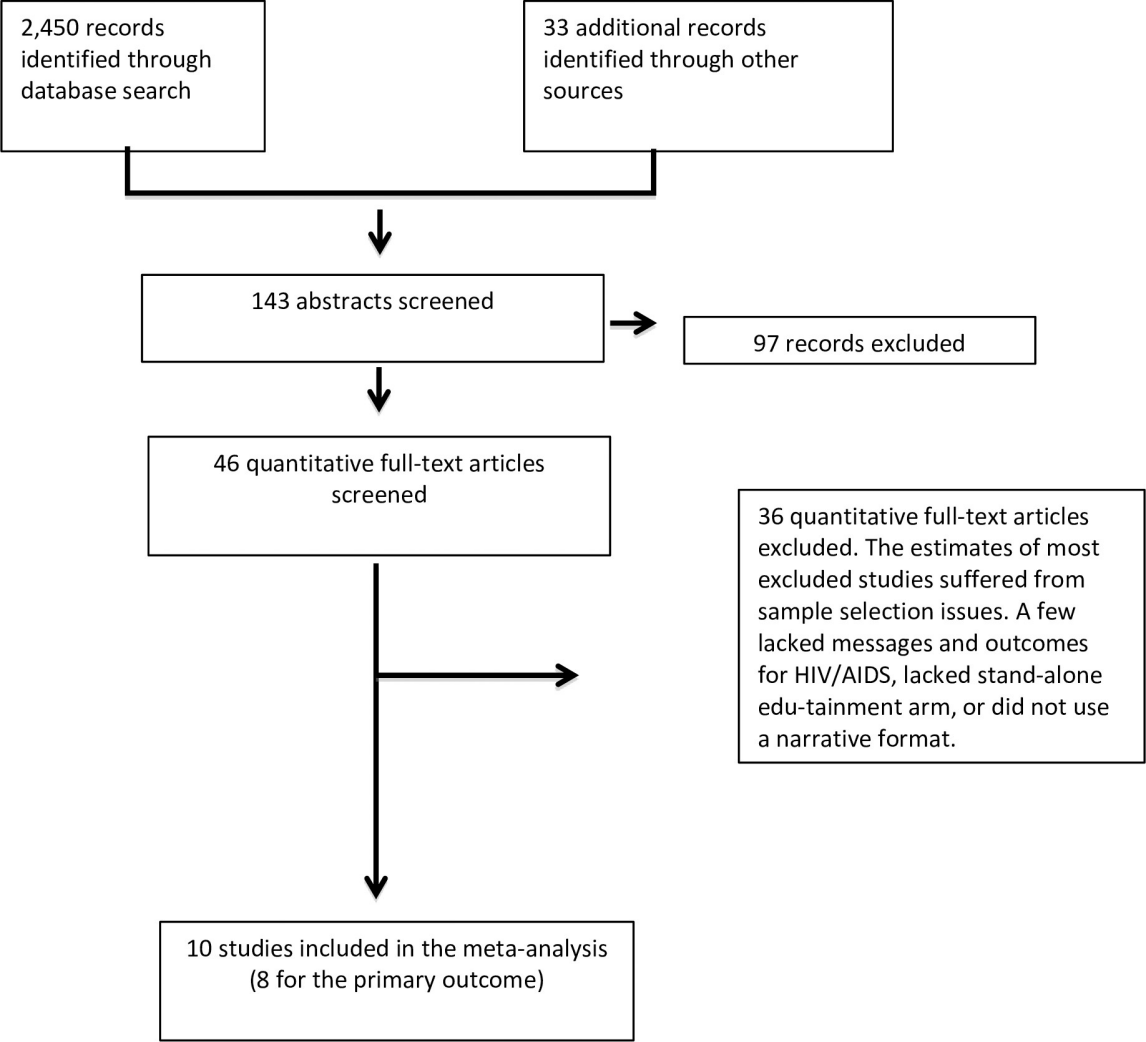
Flow diagram of study selection process, 1985–2017.

See Fig 1. Our electronic search strategy yielded 2,450 records. Forty-six studies were deemed as quantitative evaluations that suggested causal analysis. Of these, the complete article was retrieved and assessed. Thirty-six studies did not meet our inclusion criteria. S2 List and S2 Table respectively provide the list and the reasons why they these studies were excluded. Thus, the meta-analysis is based on 10 studies. Eight studies included behavior outcomes and two studies only secondary outcomes (knowledge and attitudes). Study overlap with previous systematic reviews of mass media interventions was low: two of the ten included studies [54, 55] were covered *in at least one* of the previous reviews.

### Key features of included studies

Table 1 presents information regarding the studied populations and the interventions of the 10 studies. S3 Table provides information regarding their study designs and their outcomes. Included studies were reported between 1988 and 2017, with half of them reported 2011 onwards. Five studies took place in the US and five in Sub Saharan Africa. The country’s HIV prevalence rate when the studies took place varied between 0.6 and 7.0 percent. The unweighted average age across studies was 19.3 years. Most included studies took place in urban settings. The studied interventions included TV screenings (n = 5), TV broadcasts (n = 1), radio broadcasts (n = 1), videos delivered through smart phones or personal computers (n = 2), and comics (n = 1). While the experimental studies had control over program exposure, the non-experimental (field) evaluations were based on potential exposure to public broadcasts. Controlled exposure was usually under one hour, while potential exposure was above 40 hours. As effectiveness trials, the meta sample was large with a total of 23,476 participants (a median of 902 participants per study). The endline outcomes were measured between two weeks and one year, with six studies measuring outcomes after three months or more.

**Table 1:** Descriptive characteristics of 10 included studies.

Study	Study population Characteristics	Sexual behavior included	Study parti-cipants	Intervention and Exposure time	Follow up
Banerjee *et al*. 2017 [56]	Men and women (average age 20.5) in urban Nigeria	Yes	4,986	Community screenings of edu-tainment TV drama season, MTV Shuga (2.5 hours)	6 months
Dupas *et al*. 2011 [4]	Eight-grade school female students (average age 15.3) in rural Kenya	Yes	6,196	UNICEF edu-tainment comic movie “Sara: The Trap” (10 minutes), combined with HIV relative risk information	9–12 months
Dupas *et al*. 2017 [57]	Eight-grade school female students (average age 15.5) in rural and urban Cameroon	Yes	700	Same than Dupas 2011. This complex intervention was experimentally added to a broader intervention	9–12 months
Jones *et al*. 2012 [58]	African American women (average age 22) in urban US	Yes	902	Twelve 18-minute episodes of edu-tainment TV drama “Love, Sex, and Choices”, streamed through smart phones (3.5 hours)	3 months
Kearney *et al*. 2015 [33]	Female teenagers (average age 17) in rural and urban US	Yes	4,100*	TV broadcasts of reality commercial series “MTV 16 and Pregnant” (potential exposure of 45 hours)	3 months
Milleliri *et al*. 2003 [54]	Male and female students (average age 19.1) in peri-urban Gabon	No	594	Cartoon comic about risks of unprotected.	3 weeks
Moyer-Gusé *et al*. 2010 [39]	Male and female college students (average age 20) in urban US	Yes	360	Episode screening of TV commercial drama “The OC” (30 mins)	2 weeks
Solomon *et al*. 1988 [59]	African American male patients of STI clinic (average age 23) in urban US	Yes	902	Educational video clip in a drama-format on top of counselling about importance of STI treatment (10 mins)	2 weeks
Vaughan *et al*. 2000 [55]	Men and women (average age 31) in rural Tanzania	Yes	1,230	Bi-weekly radio broadcasts of edu-tainment show “Let's Go with the Times” for two years (potential exposure of 50 hours)	3 months
Wang *et al*. 2016 [60]	Female undergraduate Latina students (average age 20.7) in urban US	No	62	Online exposure to TV show East Los High (potential exposure of one hour).	2 weeks

*Number of participants is larger as the number represents instead development media markets (205, across 20 quarters for a total of 4,100 observations).

Regarding the study design and quality, eight studies were experimental and two used quasi-experimental methods. Only one of the ten studies was not properly powered [60]. The support for risk-of-bias judgements is presented in S3 Table. The most common risk-of-bias was not fully blinding participants and assessors, followed by incomplete outcome data at follow-up. The overall risk of bias is considered low for most studies.

The data suggests that publication bias is likely to be present, though it should not substantially change the estimates of our effect sizes. On the one hand, the funnel plot shows a higher concentration of studies with positive results (See S1 Fig). On the other hand, Egger's Test of the Intercept [Egger’s Intercept (2 tails) = 1.54, 95% CI = -0.34–3.44, t = 1.99, df = 6, p = 0.10] and Rosenthal’s fail-safe (N = 45) suggest no publication bias. The latter test means that we would need to locate and include 45 null studies (or 5.6 studies null studies per included study) for the combined 2-tailed p-value to exceed 0.05. Despite the large meta-sample (n = 23,476), the results of this review should be read cautiously given the small number of studies in the primary analysis (n = 8).

### Measures of risky sexual behavior

**Fig 2:**
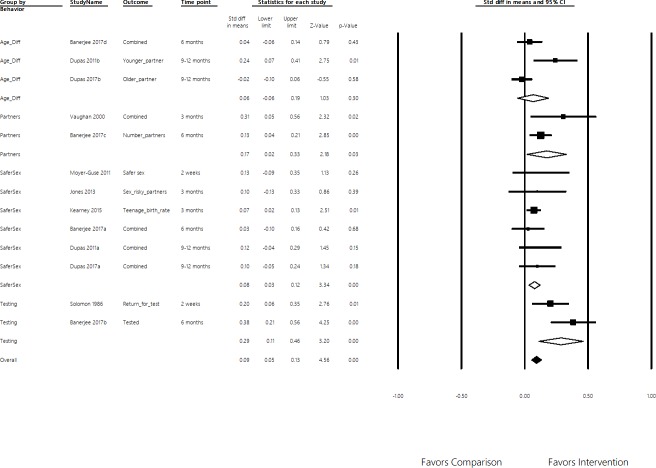
Effects on four sexual behaviors. Age-gap, number of sexual partners, unprotected sex, and STI testing and follow up. Positive values represent safer sexual practices. Standardized Mean Differences of Behavior Outcomes, 95% Confidence Interval.

Five studies collected objective behavior measures, usually in addition to the self-reported data. Objective measures included teenage birth rates, information-seeking behaviors, and percent of patients that got tested for HIV. Most self-reported items were similar to HIV questions used by the Demographic and Health Surveys program [61], a standardized health survey conducted in over one hundred countries. Fig 2 presents the effect sizes for the four behavior categories, and the overall pooled effect. S4 Table lists the SMD effects for individual behavior outcomes.

### Effectiveness on safer sexual practices

Overall, individuals that were exposed to entertainment education narratives were more likely than comparison participants to engage in safer sexual practices. We observed clinically small (SMDs between 0.08 and 0.29) but statistically significant effects for three of the four measured sexual behaviors. Our large meta-sample helped detect these small effects, for which individual studies were often not powered to do. Three studies measured impacts on inter-generational sex, through the age-gap of sexual partners [4,56,47]. The effect size was not significant (SMD = 0.06, 95% CI = -0.06–0.19, four effect sizes), though study heterogeneity was high (Q = 7.5, df(Q) = 2, I^2^ = 73.3). Two studies measured effects on the number of sexual partners [56,55]. The effect size was significant (SMD = 0.17, 95% CI = 0.02–0.33, three effect sizes), though study heterogeneity was high (Q = 1.7, df(Q) = 1, I^2^ = 40.3). Six studies measured unprotected sex practices [4,33,39,56,57,58], defined by this review as measures related condom use and pregnancy. The effect size for unprotected sex was significant (SMD = 0.08, 95% CI = 0.03–0.12, nine effect sizes), with no statistical heterogeneity observed (Q = 1.2, df(Q) = 5, I^2^ = 0). Two studies measured STI testing and management follow up practices [56,59], finding a significant effect (SMD = 0.29, 95% CI = 0.11–0.46, two effect sizes). High heterogeneity was observed (Q = 2.4, df(Q) = 1, I^2^ = 57.9). The pooled effect of the four behavior outcomes was small and significant (SMD = 0.09, 95% CI = 0.06–0.12, eighteen effect sizes). Though the pooling of these different behaviors may be difficult to conceptually justify, this pooled approach is used in sensitivity tests and subgroup analysis given the limited number of studies. Sensitivity tests did not reveal single studies exerting influence on the overall effect size or effects changing by removing studies assessed at high risk of bias. The pooled effect did not differ when pooling all behavior outcomes using a random-effects model.

### Subgroup analysis

Because factors co-vary across subgroups in meta-analyses, the limited number of included studies prevented conducting a moderators analysis related to study-design, key subgroups and intervention characteristics. We present the moderation effects of the timing of the post-intervention survey due to its clearer theoretical underpinnings (i.e., we would expect program effects to diminish with time). The timing of the post-intervention data collection did not moderate outcomes, providing indicative evidence of medium-term behavior change. A meta-regression with respect to the timing of the follow up survey finds a negative and but not significant relation (Q = 1.19, df(Q) = 1, p = 0.27). The graph of this meta-regression can be found in S2 Fig.

### Knowledge and attitudes

**Fig 3:**
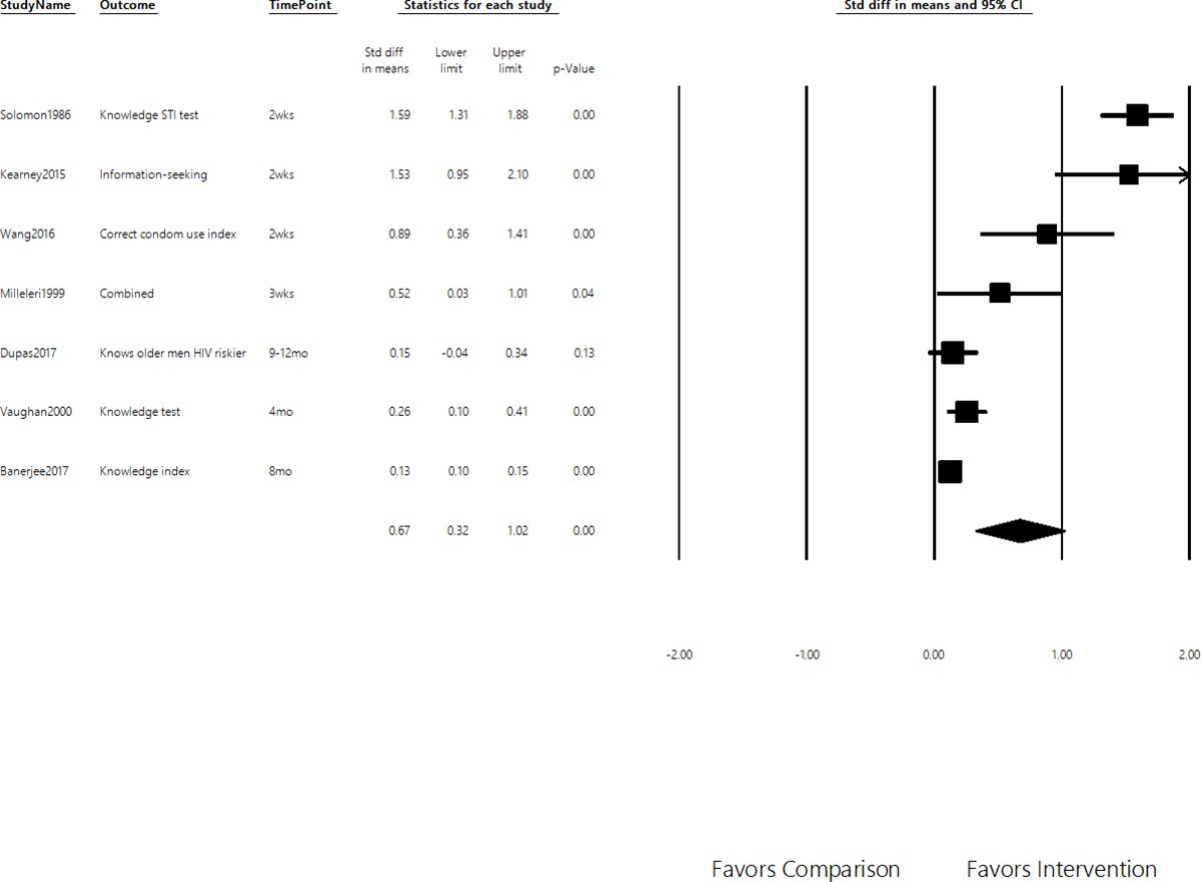
Effects on knowledge. Positive values represent intended effects. Standardized Mean Differences of Secondary Outcomes, 95% Confidence Interval.

Ten knowledge outcomes were retrieved from seven studies. Measurements for most studies were comprehensive, though a few were restricted to measuring narrow outcomes related to program messages. A medium and significant effect was found for knowledge outcomes (SMD = 0.67, 95% CI = 0.32–1.02). As seen in Fig 3, the effect size declined with the timing of the follow-up survey. This time-decaying relationship for knowledge outcomes is confirmed by the subgroup analysis. The effect size for studies that collected post-intervention data *after* a month (SMD = 0.15, 95% CI = 0.08–0.22) was approximately a fifth of the effect size of studies that measured effects *within* a month (SMD = 1.14, 95% CI = 0.61–1.68), with the difference in effect size being statistically significant (p<0.01).

**Fig 4:**
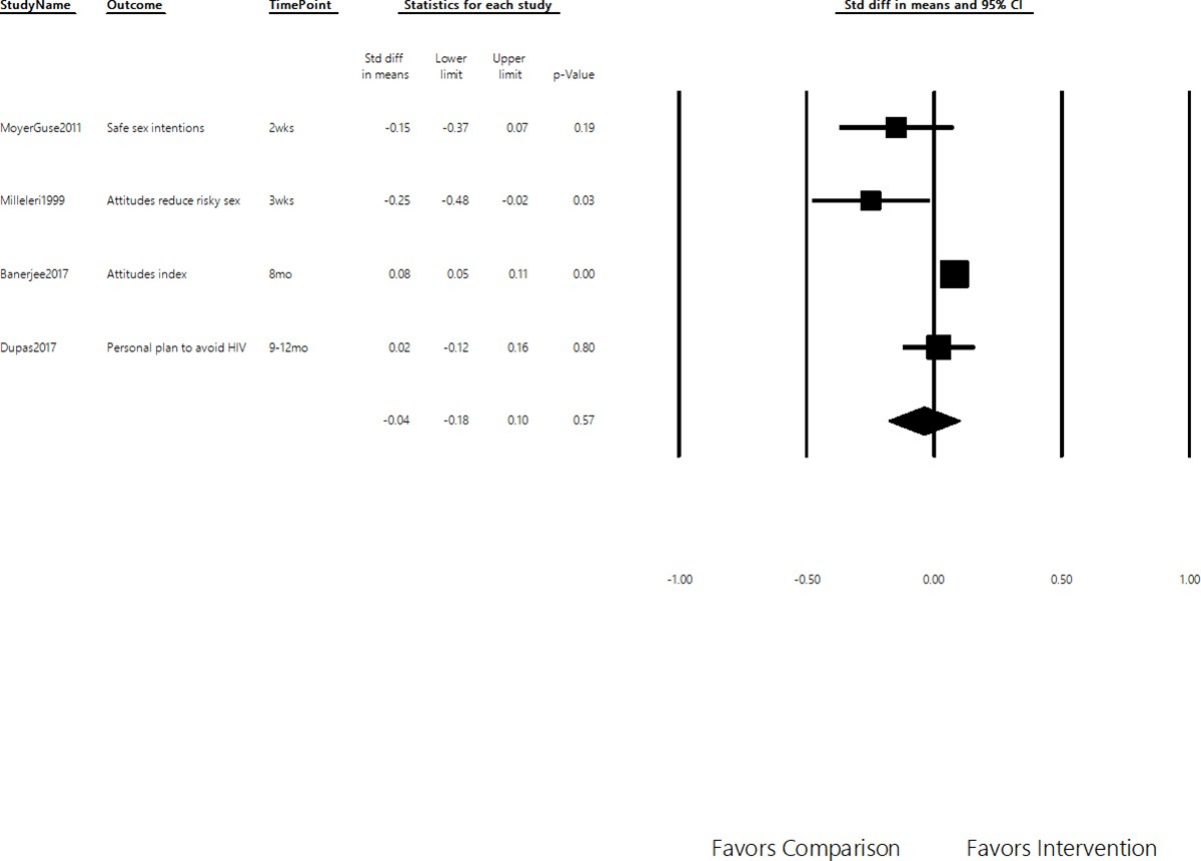
Effects on attitudes. Positive values represent intended effects. Standardized Mean Differences of Secondary Outcomes, 95% Confidence Interval.

Four attitudes outcomes were retrieved from four studies. All included studies measured prevention intentions, with one study [56] also measuring attitudes towards HIV testing and people living with HIV/AIDS. As seen in Fig 4, while no effects were observed for the pooled estimate, small negative effects were observed for the studies that measured outcomes within a month (SMD = 0.20, 95% CI = -0.36–0.04) and small positive effects when attitudes were measured after a month (SMD = 0.08, 95% CI = 0.05–0.10). The contradictory finding of the former is consistent with several excluded studies that collected data immediately after program exposure. Those studies found significant negative effects on behavioral intensions and attitudes. This may suggest self-reported biases driving this result rather than actual negative short-term effects on attitudes [62].

### Quality of the evidence

Using the Grading of Recommendations Assessment, Development, and Evaluation (GRADE) criteria [63], the quality of the evidence was considered moderate for inter-generational sex and unprotected sex; and low for number of sexual partners and STI testing and management. A “moderate” quality rating suggests further research is likely (very likely for a “low” rating) to have an important impact on our confidence in the effect size estimate. For knowledge and attitudes, it was rated high and very low respectively. See S5 Table for the GRADE Summary of Findings table. These ratings may be conservative, as recent research [64] shows that GRADE guidelines rarely give high ratings to complex and field interventions.

## Discussion

This review found that entertainment education narratives were effective in promoting HIV testing and reducing risky sex among 15-24-year-old youth. Results were robust to a series of methodological decisions. Behavioral effects persisted for several months after program exposure, with no distinguishable differences between short and medium-term effects. Behavioral effects were small (SMD between 0.08 and 0.29) and were approximately half the size reported in previous meta-analyses of mass media interventions [42,43]. However, a direct comparison is not possible given important differences in terms of populations, outcomes, study designs and interventions. The use of study participants from the general population and the use of effectiveness trials mitigate concerns regarding the scalability of entertainment education narratives [65]. Not clearly reporting blinding and selective reporting of outcomes, were the main quality concerns of included studies. Despite their relatively large sample sizes and high-quality designs, the limited number of studies remains the main limitation of this meta-analysis.

Systematic reviews of HIV behavior change find mixed effects on reducing risky sex and HIV infections, especially in developing countries [12,10]. Despite the evidence of small size effects, the marginal cost for reaching individuals should be lower above a certain “audience” threshold when compared to more resource-intensive or face-to-face interventions. High-quality entertainment shows are often consumed by millions of individuals. For example, one of the studied interventions [56] is broadcast in over seventy countries. Most included studies that found positive effects in knowledge outcomes also found behavior effects, which suggests that knowledge is a necessary but not a sufficient condition for behavior change. The ambiguous impacts on attitudes may be partly explained by measurement issues, including reporting biases triggered by short-term surveys that make salient program objectives [62]. Data limitations prevented carrying out a subgroup analysis for key subgroups and intervention characteristics.

Although the number of higher-quality evaluations has been slowly growing in the last decade, important knowledge gaps remain. More experimental research is needed, particularly in Europe, Latin America and South Asia. This review could not find studies in these regions that met our inclusion criteria. The question of program effectiveness per HIV prevalence or country-income levels remains. Moderators research is needed to better understand the dose-response relationship and the minimum amount of investments needed to expect effects in the long-term. Studies should be powered to study impacts for different subgroups [66,6], including gender, urban/rural location and audiences exposure to media. Of particular policy interest is understanding program effects on individuals that repeatedly engage in risky sexual behaviors as these individuals have the potential to infect a larger number of individuals [15]. Researchers may also experimentally investigate the effectiveness of different media outlets (i.e., TV, radio, smartphones); and study the additional effectiveness of individual components of comprehensive media campaigns [67]. Given the presence of social desirability bias in HIV research, future research should complement survey work with objective measurements, such as STI biomarkers.

With the rapid expansion of mass media in developing countries, entertainment education offers an important opportunity to promote positive behavior [68,69]. As development partners are increasingly demanding higher quality evaluations [70, 71], and with competing HIV prevention approaches producing experimental evidence of their investments [72,73], entertainment education needs to invest in stronger evaluation research. The evidence base remains mostly qualitative or correlational. Credible program effects may help future investments towards this promising communications approach.

## Supporting information

S1 Table PRISMA checklist. (DOCX)

S2 Table Intervention and reason for exclusion. (DOCX)

S3 Table Characteristics of included studies and support for risk of bias assessment. (DOCX)

S4 Table Effects on individual behavior outcomes. (DOCX)

S5 Table Summary of findings for the main comparison. (DOCX)

S1 Fig Funnel plot for primary analysis (n = 8). (DOCX)

S2 Fig Meta-regression effect size vs timing of the follow up. (DOCX)

S1 List MEDLINE search strategy. (DOCX)

S2 List List of 36 studies excluded from the meta-analysis. (DOCX)
