# Supplementary material for: The effectiveness of using entertainment education narratives to promote safer sexual behaviors of youth: A meta-analysis, 1985-2017
Source: PLoS One. 2019 Feb 12;14(2):e0209969. doi: 10.1371/journal.pone.0209969 (PMC6372167; doi:10.1371/journal.pone.0209969)
Supplement: S2 Table — (DOCX) [file pone.0209969.s002.docx]

**S2 Table. Intervention and Reason for Exclusion**

| **Study** | **Intervention** | **Reason for exclusion** |
| --- | --- | --- |
| Anita 2008 | Effects of TV on teenage pregnancies | The study compares viewers vs. non-viewers without properly addressing selection issues. |
| Arroyave 2008 | Effects of edutainment dramas on sexual attitudes and intentions. | RCT. Based on exit surveys collected immediately after exposure. |
| Brodie 2001 | TV broadcasts of “ER” in the USA with HIV messages | The study sample included a wide range of ages, with the youngest cohort set at 18-30 years of age. |
| Boulay 2002 | Radio drama in Nepal with family planning focus. | The study compares listeners vs. non-listeners with only post-intervention data. |
| Collins 2003 | TV sitcom episode in the USA with condom efficacy message | The study compares viewers vs. non-viewers with only post-intervention data. |
| Do 2006 | TV drama in Bangladesh with family planning focus. | The study uses propensity score matching with 14 control variables only using a large post-intervention survey. Selection bias may persist. |
| Farr 2005 | TV program about HIV/AIDS in Ethiopia | The study compares viewers with non-viewers with limited controls. Selection bias may persist. |
| Geary 2007 | Evaluation of HIV mass media campaigns in Nepal, Brazil and Senegal | The campaign included a series of media components. The study collects data before and after data, comparing consumers vs non-consumers. |
| Jadranin 2015 | Movie with HIV/AIDS messages with Serbian soldiers | Before-after comparison that lacks a control group. |
| Jensen 2009 | Community cable access (main medium for soap operas in India) in India | Family planning outcomes (number of children and birth spacing at the individual level) lacking high-risk sexual behaviors and not provided for youth. |
| Karlyn 2001 | Radio drama in Mozambique aimed to prevent STI | The study compares listeners vs. non-listeners with only post-intervention data. |
| Keating 2006 | Evaluation of HIV mass media campaigns in Nigeria | The campaign included a series of media components. The study collects data before and after data, comparing consumers vs non-consumers. |
| Kuhlmann 2008 | Radio drama in Botswana with PMTCT focus | The study compares listeners vs. non-listeners with only post-intervention data. |
| La Ferrara 2012 | Commercial TV dramas in Brazil | The study did not focus on HIV messages and youth. |
| Lapinski 2008 | Short film about HIV stigma in Nigeria | RCT. Based on exit surveys collected immediately after exposure. |
| Middlestadt 1995 | Radio campaign in St. Vincent and the Grenadines with HIV/AIDS messages | The study compares listeners vs. non-listeners with only post-intervention data. |
| Mohammed 2001 | Radio drama in Tanzania with family planning focus | The study compares listeners vs. non-listeners with only post-intervention data. |
| Moyer 2011b | TV drama in the USA with safe sex messages | RCT. The treatment arm includes post-screening discussions. |
| O’Donnell 1995 | Video-based interventions promoting condom use in STD clinic | RCT. The intervention video-based patient education program and not an entertainment-education drama |
| O'leary 2007 | TV drama in Botswana with HIV de-stigmatization focus | The study compares viewers vs. non-viewers with only post-intervention data. |
|  |  |  |
| Pappas-DeLuca 2008 | Radio drama in Botswana with HIV prevention focus. | The paper studies associations between program exposure and outcomes, without attempting to construct a control group. |
| Peltzer 2003 | TV drama in South Africa with HIV and condom messages | The TV drama was bundled with other interventions. The study is based on post-intervention data only. |
| Peltzer 2004 | TV drama in South Africa with HIV and condom messages | The TV drama was bundled with other interventions. The study is based on post-intervention data only. |
| Ramirez 2014 | Short film for US Latino communities with Stigma towards gay and bisexual | Before-after study without a comparison group |
| Rogers 1999 | Radio drama in Tanzania with family planning messages | The evaluation of this intervention is presented by family planning outcomes (Rogers 1999) and high-risk sexual outcomes (Vaughn 1999). The latter is an included study. |
| Sahka 2013 | TV drama in Iran targeted to FSW | Before-after study that bundled several interventions to the educational movie |
| Shapiro 2006 | TV drama in Cote d’Ivoire with HIV focus | The study compares viewers vs. non-viewers with only post-intervention data. |
| Silvestre 2015 | Evaluation of HIV mass media campaigns in Nepal, Brazil and Senegal | The campaign included a series of media components. The study compares consumers vs non-consumers with only post-intervention data. |
| Smith 2007a | Radio drama in Ethiopia with HIV prevention focus | The study compares listeners vs. non-listeners with only post-intervention data. |
| Storey 1999 | Radio drama in Nepal with family planning messages | Study targets married women and only includes family planning outcomes. |
| Vaughan 2000b | Radio drama in St Lucia with HIV prevention focus | The study is underpowered and selection bias is likely to be significant. Although study collected pre and posttest data for listeners and non-listeners, limited attempts to control for characteristics that could affect listenership and key outcomes. |
| Vernon 2007 | Radio drama in Colombia with family planning focus | The study is based on a before-after analysis without a comparison group. |
| Whittier 2005 | TV sitcom episode in the USA with STI focus for MSM | The study compares viewers vs. non-viewers with only post-intervention data. |
| Yoder 2006 | Radio drama in Zambia with HIV prevention focus | The study collects data before and after data, comparing listeners to non-listeners. Selection bias may persist. |
