# Supplementary material for: The effectiveness of using entertainment education narratives to promote safer sexual behaviors of youth: A meta-analysis, 1985-2017
Source: PLoS One. 2019 Feb 12;14(2):e0209969. doi: 10.1371/journal.pone.0209969 (PMC6372167; doi:10.1371/journal.pone.0209969)
Supplement: S3 Table — (DOCX) [file pone.0209969.s003.docx]

**S3 Table. Characteristics of Included Studies and Support for Risk of Bias Assessment**

| \| **Banerjee 2017** \| \| --- \| |  |  |
| --- | --- | --- | --- |
| Methods | CRCT | |
| Outcomes | Knowledge (about ARVs and of waiting period), Sexual behaviors (used condom in last sexual intercourse, reduction in number of current sexual partners, got tested in last six months). | |
| **Bias** | **Authors' judgement** | **Support for judgement** |
| Random sequence generation (selection bias) | Low risk | Community centers were randomly allocated with computer random number generator. |
| Allocation concealment (selection bias) | Low risk | Community centers centrally allocated by the research team. |
| Blinding of participants and personnel (performance bias) | High risk | Community-level randomization with buffer areas minimize performance bias. |
| Blinding of outcome assessment (detection bias) | Low risk | In addition to self-reported data, the study collects objective measures for HIV testing. The post-intervention survey was conducted by a survey firm independent of the implementing NGO and six months after exposure, which further reduces concerns for detection bias. |
| Incomplete outcome data (attrition bias) | Low risk | The study’s attrition rate is 3 percent, with no systematic differences in overall rates or reasons between the treatment and the control groups. |
| Selective reporting (reporting bias) | Low risk | Reporting bias is unlikely given that the study protocol pre-specifies primary and secondary outcomes and the study reports results for all these outcomes, in addition to constructing aggregated indexes. |
| Other bias | Low risk | Baseline balance, standard errors were properly adjusted in the analysis and recruitment for the treatment and control groups was conducted in a similar way. |
|  |  |  |
| **Dupas 2011** |  |  |
| Methods | CRCT | |
| Outcomes | Sexual behaviors (teenage pregnancy, used condom last sexual intercourse, age difference with baby’s father) | |
| **Bias** | **Authors' judgement** | **Support for judgement** |
| Random sequence generation (selection bias) | Low risk | Schools were randomly allocated with computer random number generator. |
| Allocation concealment (selection bias) | Low risk | Schools centrally allocated by the research team. |
| Blinding of participants and personnel (performance bias) | Low risk | School-level randomization with buffer areas minimize performance bias. |
| Blinding of outcome assessment (detection bias) | Low risk | The primary outcome, teenage pregnancy, is objectively corroborated by the enumerators. |
| Incomplete outcome data (attrition bias) | Low risk | Attrition rates are under 2 percent, with no systematic differences between treatment and control groups. |
| Selective reporting (reporting bias) | Low risk | All primary outcomes are reported. |
| Other bias | Low risk | No other issues were found. |
|  |  |  |
| \| **Dupas 2017** \| \| --- \| |  |  |
| Methods | CRCT | |
| Outcomes | Sexual behaviors (teenage pregnancy, used condom last sexual intercourse, age difference with baby’s father) | |
| **Bias** | **Authors' judgement** | **Support for judgement** |
| Random sequence generation (selection bias) | Low risk | Schools were randomly allocated with computer random number generator. |
| Allocation concealment (selection bias) | Low risk | Schools centrally allocated by the research team. |
| Blinding of participants and personnel (performance bias) | Low risk | School-level randomization with buffer areas minimize performance bias. |
| Blinding of outcome assessment (detection bias) | Low risk | The primary outcome, teenage pregnancy, is objectively corroborated by the enumerators. |
| Incomplete outcome data (attrition bias) | Low risk | Attrition rates are under 2 percent, with no systematic differences between treatment and control groups. |
| Selective reporting (reporting bias) | Low risk | All primary outcomes are reported. |
| Other bias | Low risk | No other issues were found. |
|  |  |  |
| \| **Jones 2012** \| \| --- \| |  |  |
| Methods | Quasi-experimental (ITS) | |
| Outcomes | Knowledge (Knew about prevention options after unprotected sex) and sexual behaviors (Vaginal episode equivalent with high risk partners) | |
| **Bias** | **Authors' judgement** | **Support for judgement** |
| Random sequence generation (selection bias) | Low risk | Study participants were randomly allocated with computer random number generator. |
| Allocation concealment (selection bias) | Low risk | Group assignment placed into sealed envelopes. |
| Blinding of participants and personnel (performance bias) | Unclear risk | Staff was not blinded though the mobile-based intervention did not require participants to interact with each other. |
| Blinding of outcome assessment (detection bias) | Low risk | Though based on self-reported data, the study uses audio-computer assisted self-interviews to minimize detection bias. |
| Incomplete outcome data (attrition bias) | High risk | 32/149 and 25/146 missing from intervention and control groups respectively. |
| Selective reporting (reporting bias) | Unclear risk | The study lacked a research protocol though main indicators seem to be reported. |
| Other bias | Low risk | No other issues were found. |
|  |  |  |
| \| **Kearney 2015** \| \| --- \| |  |  |
| Methods | Quasi-experimental (ITS) | |
| Outcomes | Knowledge-seeking behavior (Google searches for how to get birth control pills) and Sexual behavior (teenage pregnancy). | |
| **Bias** | **Authors' judgement** | **Support for judgement** |
| Random sequence generation (selection bias) | Unclear risk | The study is not experimental, though it provides a series of statistical tests to validate the used instrumental variable strategy. |
| Allocation concealment (selection bias) | Low risk | Natural experiment |
| Blinding of participants and personnel (performance bias) | Low risk | The study relied on district-level data and did not interact with study participants. |
| Blinding of outcome assessment (detection bias) | Low risk | All outcomes are measured through administrative and independent data. |
| Incomplete outcome data (attrition bias) | Low risk | Only one of 205 Designated Market Areas was removed from a 20-quarter panel. |
| Selective reporting (reporting bias) | Low risk | Though study seemed to have lacked a research protocol, the main outcomes are reported. |
| Other bias | Low risk | No other issues were found. |
|  |  |  |
| \| **Milleleri 1999** \| \| --- \| |  |  |
| Methods | CRCT | |
| Outcomes | Knowledge (testing and transmission mechanisms) and attitudes (ability to convince peers) | |
| **Bias** | **Authors' judgement** | **Support for judgement** |
| Random sequence generation (selection bias) | Unclear risk | Unclear how schools were randomly allocated. |
| Allocation concealment (selection bias) | Low risk | The research team centrally allocated schools. |
| Blinding of participants and personnel (performance bias) | Low risk | School-level randomization with buffer areas minimize performance bias. |
| Blinding of outcome assessment (detection bias) | Low risk | Knowledge questions less likely to suffer from detections bias. |
| Incomplete outcome data (attrition bias) | High risk | Attrition rates above ten percent. |
| Selective reporting (reporting bias) | High risk | The study lacked a research protocol, with key outcomes not reported. |
| Other bias | Unclear risk | The study does not provide evidence regarding pre-intervention balance. |
|  |  |  |
| \| **Moyer-Guse 2010** \| \| --- \| |  |  |
| Methods | RCT | |
| Outcomes | Sexual behavior (use of birth control) | |
| **Bias** | **Authors' judgement** | **Support for judgement** |
| Random sequence generation (selection bias) | Low risk | Study participants were randomized. |
| Allocation concealment (selection bias) | Unclear risk | Anonymity codes were used to match participants. However, students may have known each other making concealment difficult. |
| Blinding of participants and personnel (performance bias) | High risk | The lab setting, and watching the intervention in group, probably triggered Hawthorne effects. |
| Blinding of outcome assessment (detection bias) | High risk | Self-reported data that could be linked with the intervention objective. |
| Incomplete outcome data (attrition bias) | Low risk | The reported attrition rate is 4 percent, with no systematic differences between groups. |
| Selective reporting (reporting bias) | Low risk | The study reports all primary and secondary outcomes. |
| Other bias | High risk | Self-reported data collected immediately and two weeks after exposure. The former probably primed study participants’ responses for the latter, which is used in this meta-analysis. |
|  |  |  |
| \| **Solomon 1988** \| \| --- \| |  |  |
| Methods | RCT | |
| Outcomes | Sexual behavior (Notification of sex partners, return for test of cure within required two weeks) | |
| **Bias** | **Authors' judgement** | **Support for judgement** |
| Random sequence generation (selection bias) | Unclear risk | Unclear how patients were randomized. |
| Allocation concealment (selection bias) | Low risk | Probably took measures to conceal, allocation given the lab-setting of the study. |
| Blinding of participants and personnel (performance bias) | High risk | The clinicians provided the edutainment intervention on top of the business as usual intervention. |
| Blinding of outcome assessment (detection bias) | High risk | Self-reported data collected immediately and two weeks after exposure. The former probably primed study participants’ responses in the latter, which is used in this meta-analysis. |
| Incomplete outcome data (attrition bias) | Low risk | The study’s attrition rate is 4 percent. |
| Selective reporting (reporting bias) | Low risk | The study lacked a protocol, though main outcomes seem to be reported. |
| Other bias | Low risk | No other issues were found. |
|  |  |  |
| **Vaughn 2000** |  |  |
| Methods | Quasi-experimental (Difference-in-difference) | |
| Outcomes | Sexual behavior (reduction number sexual partners in previous year). Other behavior outcomes were excluded due to important baseline imbalances. | |
| **Bias** | **Authors' judgement** | **Support for judgement** |
| Random sequence generation (selection bias) | High risk | Control group comes from only one region. |
| Allocation concealment (selection bias) | Low risk | Centrally allocated by the research team. |
| Blinding of participants and personnel (performance bias) | Low risk | The non-invasive nature of the study (small number of surveys conducted for a popular radio broadcast) and the region-level randomization minimizes performance bias. |
| Blinding of outcome assessment (detection bias) | Low risk | Repeated cross sectional minimize survey effects. |
| Incomplete outcome data (attrition bias) | Low risk | The study is based on annual repeated cross-sectional surveys. |
| Selective reporting (reporting bias) | High risk | Due to important baseline imbalances, the meta-analysis could only use some of the outcomes. |
| Other bias | High risk | Important baseline imbalances for several (excluded) outcomes. |
|  |  |  |
| \| **Wang 2016** \| \| --- \| |  |  |
| Methods | RCT | |
| Outcomes | Knowledge (correct condom use) and Attitudes (importance of HIV testing). Behavior outcomes (condom use in last instance of sexual intercourse) excluded as not powered for it. | |
| **Bias** | **Authors' judgement** | **Support for judgement** |
| Random sequence generation (selection bias) | Low risk | Study participants were randomly allocated with computer random number generator. |
| Allocation concealment (selection bias) | Low risk | Probably took measures to conceal, allocation given the lab-setting of the study. |
| Blinding of participants and personnel (performance bias) | High risk | Study participants were selected from a broader project that had surveyed these participants. |
| Blinding of outcome assessment (detection bias) | High risk | Self-reported panel data collected immediately and two weeks after exposure. The former probably primed study participants’ responses in the two-week follow up, which is used in this meta-analysis. |
| Incomplete outcome data (attrition bias) | High risk | Test information suggest attrition rates above 10 percent. |
| Selective reporting (reporting bias) | Unclear risk | The research protocol was not found, with some results not fully reported. |
| Other bias | Low risk | No other issues were found. |
