# Supplementary material for: The effectiveness of using entertainment education narratives to promote safer sexual behaviors of youth: A meta-analysis, 1985-2017
Source: PLoS One. 2019 Feb 12;14(2):e0209969. doi: 10.1371/journal.pone.0209969 (PMC6372167; doi:10.1371/journal.pone.0209969)
Supplement: S4 Table — (DOCX) [file pone.0209969.s004.docx]

**S4 Table. Effects on Individual Behavior Outcomes**

| **Behavior outcome and Study Name** | **Sex** | **Behavior outcome** | **SMD** | **s.e.** | **Lower limit** | **Upper limit** | **Z-value** | **p-value** |
| --- | --- | --- | --- | --- | --- | --- | --- | --- |
| **Age-difference with sexual partner** | | | | | | | | |
| Dupas 2011 | F | Age-gap | 0.24 | 0.09 | 0.07 | 0.41 | 2.75 | 0.01 |
| Banerjee 2017 | M | Age-gap | 0.04 | 0.05 | -0.06 | 0.13 | 0.72 | 0.47 |
| Banerjee 2017 | F | Age-gap | 0.04 | 0.05 | -0.06 | 0.14 | 0.86 | 0.39 |
| Dupas 2017 | F | Age-gap | -0.02 | 0.04 | -0.10 | 0.06 | -0.55 | 0.58 |
| **Number of sexual partners** | | | | | | | | |
| Banerjee 2017 | F+M | Number sexual partners | 0.13 | 0.04 | 0.04 | 0.21 | 2.85 | 0.00 |
| Vaughan 2000 | M | Number sexual partners | 0.38 | 0.15 | 0.08 | 0.67 | 2.48 | 0.01 |
| Vaughan 2000 | F | Number sexual partners | 0.23 | 0.11 | 0.02 | 0.45 | 2.16 | 0.03 |
| **Unprotected sex** | | | | | | | | |
| Moyer-Guse 2011 | F+M | Safer sex | 0.13 | 0.11 | -0.09 | 0.35 | 1.13 | 0.26 |
| Dupas 2011 | F | Teenager childbearing | 0.06 | 0.03 | 0.00 | 0.12 | 1.88 | 0.06 |
| Dupas 2011 | F | Condom use last event | 0.19 | 0.12 | -0.04 | 0.41 | 1.61 | 0.11 |
| Jones 2013 | F | Vaginal episode w/high-risk partner | 0.10 | 0.12 | -0.13 | 0.33 | 0.86 | 0.39 |
| Kearney 2015 | F | Teenager birth rate | 0.07 | 0.03 | 0.02 | 0.13 | 2.51 | 0.01 |
| Banerjee 2017 | M | Condom use last event | 0.06 | 0.07 | -0.07 | 0.19 | 0.86 | 0.39 |
| Banerjee 2017 | F | Condom use last event | 0.00 | 0.06 | -0.13 | 0.12 | -0.04 | 0.97 |
| Dupas 2017 | F | Currently pregnant | 0.09 | 0.07 | -0.05 | 0.23 | 1.27 | 0.20 |
| Dupas 2017 | F | Unprotected sex last 12mon | 0.11 | 0.08 | -0.04 | 0.26 | 1.41 | 0.16 |
| **STI testing and management** | | | | | | | | |
| Solomon 1986 | M | Return for STI test of cure | 0.20 | 0.07 | 0.06 | 0.35 | 2.76 | 0.01 |
| Banerjee 2017 | F+M | Tested last 6 mon | 0.38 | 0.09 | 0.21 | 0.56 | 4.25 | 0.00 |

M=male, F=female, SMD=standardized mean difference, s.e.= standard error.
