# Supplementary material for: The effectiveness of using entertainment education narratives to promote safer sexual behaviors of youth: A meta-analysis, 1985-2017
Source: PLoS One. 2019 Feb 12;14(2):e0209969. doi: 10.1371/journal.pone.0209969 (PMC6372167; doi:10.1371/journal.pone.0209969)
Supplement: S5 Table — (DOCX) [file pone.0209969.s005.docx]

**S5 Table. Summary of Findings for the Main Comparison**

| **Entertainment-education narratives compared with no intervention or treatment as usual for reducing risky sexual behaviors** | | | | |
| --- | --- | --- | --- | --- |
| **Patient or population: Youth from the general population**  **Settings:** Audiences from mass media shows and individuals recruited in schools  **Intervention:** Entertainment-education narratives delivered in a narrative format  **Comparison:** No intervention, treatment as usual | | | | |
| **Outcomes** | **Illustrative comparative risks* (95% CI)** | **No of Participants (studies)** | **Quality of the evidence (GRADE)** | **Comments** |
|  | **Corresponding risk** |  |  |  |
|  | **Entertainment-education narratives** |  |  |  |
| **Age-Gap with sexual partners** (self-reported) pre-and post-treatment) | Across studies, the number and age of sexual partners was on average **0.06 standard deviations better** (with a 95% CI ranging from -0.06 to 0.19) than in the control condition. | 11,882  (3 studies) | ⊕⊕⊕⊝ **moderate ^3^** | No effect |
| **Number of sexual partners** (self-reported) pre-and post-treatment) | Across studies, the number and age of sexual partners was on average **0.17 standard deviations better** (with a 95% CI ranging from -0.02 to 0.33) than in the control condition. | 6,216  (2 studies) | ⊕⊕⊝⊝ **low ^1, 2^** | Small effect |
| **Unprotected sex** (self-reported and objective measures) post-treatment) | Across studies, the number and age of sexual partners was on average **0.08 standard deviations better** (with a 95% CI ranging from -0.03 to 0.12) than in the control condition. | 16,594  (6 studies) | ⊕⊕⊕⊝ **moderate ^2^** | Small effect |
| **STI testing and management follow up** (self-reported and objective measures) post-treatment) | Across studies, the number and age of sexual partners was on average **0.29 standard deviations better** (with a 95% CI ranging from -0.11 to 0.46) than in the control condition. | 5,902  (2 studies) | ⊕⊕⊝⊝ **low ^3 4^** | Small effect |
| **Knowledge of HIV and STI transmission** (test scores) pre-and post-treatment | Across studies, test scores were on average **0.67 standard deviations better** (with a 95% CI ranging from 0.32 to 1.02) than in the control condition. | 2,699  (7 studies) | ⊕⊕⊕⊕ **high** | Large effect in short-term, small effect in long-term |
| **Attitudes towards behavior intensions and people living with HIV/AIDS** (self-reported) pre-and post-treatment | **Across studies, the typical scores were on average -0.04 standard deviations worse** (with a 95% CI ranging from -0.18 to 0.10) than in the control condition. | 7,795  (4 studies) | ⊕⊝⊝⊝ **very low ^1, 3, 4^** | Methodological issues are likely driving inconsistent results. |
| **GRADE Working Group grades of evidence:**  **High quality**: Further research is very unlikely to change our confidence in the estimate of effect.  **Moderate quality:** Further research is likely to have an important impact on our confidence in the estimate of effect and may change the estimate.  **Low quality:** Further research is very likely to have an important impact on our confidence in the estimate of effect and is likely to change the estimate.  **Very low quality:** We are very uncertain about the estimate. | | | | |
| Downgraded for:  ^1^ Limitations in design.  ^2^ Indirectness of evidence.  ^3^ Unexplained heterogeneity or inconsistency of results.  ^4^ Imprecision of results. | | | | |
