# Supplementary material for: The effectiveness of using entertainment education narratives to promote safer sexual behaviors of youth: A meta-analysis, 1985-2017
Source: PLoS One. 2019 Feb 12;14(2):e0209969. doi: 10.1371/journal.pone.0209969 (PMC6372167; doi:10.1371/journal.pone.0209969)
Supplement: S1 Fig — (DOCX) [file pone.0209969.s006.docx]

**S1 Fig. Funnel Plot for Primary Analysis (n=8)**

Observed studies in blank, imputed studies in black.
