# Supplementary material for: The effectiveness of using entertainment education narratives to promote safer sexual behaviors of youth: A meta-analysis, 1985-2017
Source: PLoS One. 2019 Feb 12;14(2):e0209969. doi: 10.1371/journal.pone.0209969 (PMC6372167; doi:10.1371/journal.pone.0209969)
Supplement: S2 Fig — (DOCX) [file pone.0209969.s007.docx]

**S2 Fig. Meta-regression Effect Size vs Timing of the Follow Up**
