# Supplementary material for: The effectiveness of using entertainment education narratives to promote safer sexual behaviors of youth: A meta-analysis, 1985-2017
Source: PLoS One. 2019 Feb 12;14(2):e0209969. doi: 10.1371/journal.pone.0209969 (PMC6372167; doi:10.1371/journal.pone.0209969)
Supplement: S1 List — (DOCX) [file pone.0209969.s008.docx]

**S1 List. MEDLINE search strategy**

**HIV/AIDS Keywords**

1. HIV infections/pc
2. Sexually transmitted diseases/pc
3. Acquired Immunodeficiency Syndrome/pc
4. (HIV or AIDS).ti,ab
5. or 1-4

**Evaluation Keywords**

1. Program evaluation/
2. Randomized controlled trials/
3. Evaluation studies/
4. Random allocation/
5. Control trial.ti,ab
6. Controlled trial.ti,ab
7. (rct or rcts).ti,ab
8. Evaluation$.ti,ab
9. Impact$.ti,ab
10. (effect or effects or effective or effectiveness).ti,ab
11. or 6-15

**Intervention Keywords**

1. Intervention$.ti,ab
2. Communication$.ti,ab
3. Drama$.ti,ab
4. Edu-tainment.ti,ab
5. Edutainment.ti,ab
6. Entertainment.ti,ab
7. Film$.ti,ab
8. Mass media$.ti,ab
9. Novela$.ti,ab
10. Radio.ti,ab
11. Soap opera$.ti,ab
12. Soaps.ti,ab
13. Telenovela$.ti,ab
14. Television.ti,ab
15. TV.ti,ab
16. Youth.ti,ab
17. Young.ti,ab
18. or 17-33

**Sexual behavior Keywords**

1. Health behavior/
2. Reproductive health/
3. HIV testing/
4. Risk reducing behavior/
5. Contraception behavior/
6. Sexual behavior/
7. Sexual partners/
8. Safe sex/
9. Sexual Concurrency/
10. Unsafe sex/
11. Condom$/ut
12. Contracep$.ti,ab
13. Sex$.ti,ab
14. Stigma ti,ab
15. or 35-48
16. 5 and 16 and 34 and 49

Limits: Limited to English language

Publication limits: Multi-center study, Corrected and republished article, Evaluation studies, Journal article, Meta-analysis, Randomized controlled trial, Review literature, Technical report, Validation studies.
