# Supplementary material for: The effectiveness of using entertainment education narratives to promote safer sexual behaviors of youth: A meta-analysis, 1985-2017
Source: PLoS One. 2019 Feb 12;14(2):e0209969. doi: 10.1371/journal.pone.0209969 (PMC6372167; doi:10.1371/journal.pone.0209969)
Supplement: S2 List — (DOCX) [file pone.0209969.s009.docx]

**S2 List. List of 36 studies excluded from the meta-analysis**

Anita C, Martino S, Collins R, Elliott M, Berry S, Kanouse D, Miu A. (2008). **Does Watching Sex on Television Predict Teen Pregnancy? Findings from a National Longitudinal Survey of Youth,** *Pediatrics* **122(5):**1047-1054.

Arroyave J. Testing the effectiveness of an entertainment-education health-focused soap opera: exposure and post-discussion in Colombian young adults. Investigación & Desarrollo 2008; vol. 16, núm. 2, pp. 232-261

Boulay M, Storey JD, Sood S**. Indirect exposure to a family planning mass media campaign in Nepal.** *Journal of Health Communications* 2002; Oct-Dec 7(5):379-99.

Brodie M, Foehr U, Rideout V, Baer N, Miller C, Flournoy R, Altman D. **Communicating health information through the entertainment media: A study of the television drama ER lends support to the notion that Americans pick up information while being entertained**, Health Affairs, 20, 192–199., 2001.

Collins RL, Elliott MN, Berry SH, Kanouse EE, and Hunter SB. (2003). **Entertainment television as a healthy sex educator: The impact of condom efficacy information in an episode of Friends.** *Pediatrics,* **112,** 1115–1121.

Do M, Kinkaid L. **Impact of an Entertainment-Education Television Drama on Health Knowledge and Behavior in Bangladesh: An Application of Propensity Score Matching.** *Journal of Health Communication* 2006; **11**:301–325

Farr AC, Witte K, Jarato K, and Menard T. (2005). **The effectiveness of media use in health education: Evaluation of an HIV/AIDS radio campaign in Ethiopia.** *Journal of Health Communication*, **10(3)**, 225-235.

Geary CW, Burke HM, Castelnau L, Neupane S, Sall YB, Wong E, Tucker HT. **MTV's Staying Alive global campaign promoted interpersonal communication about HIV and positive beliefs about HIV prevention**. *AIDS Education Prevention* 2007 Feb;**19(1**):51-67.

Jadranin Z, Dedic G, Vaughan F, Grillo M, Suljagic V. **The impact of an educational film on promoting knowledge and attitudes toward HIV in soldiers of the Serbian Armed Forces.** *Vojnosanit Pregl.* 2015 Jul;**72(7):**569-75.

Jensen R, Oster E. **The Power of TV: Cable Television and Women's Status in India.** *The Quarterly Journal of Economics* 2009; MIT Press, vol. **124(3),** pages 1057-1094, August.

Karlyn A. **The impact of a targeted radio campaign to prevent STIs and HIV/AIDS in Mozambique.** *AIDS Education Prevention*. 2001 Oct; **13(5):**438-51.

Keating J, [Meekers D](http://www.ncbi.nlm.nih.gov/pubmed/?term=Meekers%20D%5BAuthor%5D&cauthor=true&cauthor_uid=16672067), [Adewuyi A](http://www.ncbi.nlm.nih.gov/pubmed/?term=Adewuyi%20A%5BAuthor%5D&cauthor=true&cauthor_uid=16672067). **Assessing effects of a media campaign on HIV/AIDS awareness and prevention in Nigeria: results from the VISION Project.** [*BMC Public Health.*](http://www.ncbi.nlm.nih.gov/pubmed/16672067) 2006 May **3**;6: 123.

Kennedy M, O'Leary A, Beck V, Pollard K, Simpson P. **Increases in Calls to the CDC National STD and AIDS Hotline Following AIDS-Related Episodes in a Soap Opera**. *Journal of Communication*, 2004; **54:** 287–301.

Kuhlmann AKS, Kraft JM., Galavotti C, Creek TL, Mooki M, and Ntumy R. (2008). **Radio role models for the prevention of mother-to-child transmission of HIV and HIV testing among pregnant women in Botswana**. *Health Promotion International*, **23(3),** 260-268.

La Ferrara E, Chong A, Duryea S. **Soap Operas and Fertility: Evidence from Brazil.** *American Economic Journal: Applied Economics* 2012; **4 (4),** 1-31.

Lapinski M, Nwulu P. **Can a Short Film Impact HIV-Related Risk and Stigma Perceptions? Results from an Experiment in Abuja, Nigeria**, *Health Communication* 2008, **23:**5,403 — 412

Middlestadt S, Fishbein M, Albarracin D et al. **Evaluating the impact of a national AIDS prevention radio campaign in St. Vincent and the Grenadines.** *J Applied Social Psychology* 1995; **25**: 21–34.

Mohammed S. **Personal communication networks and the effects of an entertainment- education radio soap opera in Tanzania.** *Journal of Health Communication,* 2001; **6(2),** 137-154.

Moyer-Gus E, Nabi R. **Explaining the Effects of Narrative in an Entertainment Television Program: Overcoming Resistance to Persuasion**. *Human Communication Research* 2010; **36** p 26–52

Moyer-Gusé E, Chung A, and Jain P. (2011). **Identification with characters and discussion of taboo topics after exposure to an entertainment narrative about sexual health.** *Journal of Communication*, **61,** 387-406.

[O'Donnell L](http://www.ncbi.nlm.nih.gov/pubmed/?term=O'Donnell%20L%5BAuthor%5D&cauthor=true&cauthor_uid=7624818), [Sandoval A](http://www.ncbi.nlm.nih.gov/pubmed/?term=San%20Doval%20A%5BAuthor%5D&cauthor=true&cauthor_uid=7624818), [Duran R](http://www.ncbi.nlm.nih.gov/pubmed/?term=Duran%20R%5BAuthor%5D&cauthor=true&cauthor_uid=7624818), [O'Donnell CR](http://www.ncbi.nlm.nih.gov/pubmed/?term=O'Donnell%20CR%5BAuthor%5D&cauthor=true&cauthor_uid=7624818). **The effectiveness of video-based interventions in promoting condom acquisition among STD clinic patients.** *Sexually Transmitted Diseases.* 1995 Mar-Apr; **22 (2):**97-103.

O'Leary A, Kennedy M, Pappas-DeLuca K, Nkete M, Beck V, Galavotti C. **Association between exposure to an HIV story line in The Bold and the Beautiful and HIV-related stigma in Botswana. *AIDS*** *Education Prevention* 2007; Jun **19(3):**209-17.

Pappas-DeLuca KA, Kraft JM, Galavotti C, Warner L, Mooki M, Hastings P, Kilmarx PH. (2008). **Entertainment-education radio serial drama and outcomes related to HIV testing in Botswana.** *AIDS Education & Prevention,* **20(6),** 486-503.

Peltzer K, Philip S. **Evaluation of HIV/AIDS prevention intervention messages on a rural sample of South African youth’s knowledge, attitudes, beliefs and behaviours over a period of 15 months.** *J Child Adolescent Mental Health* 2004; **16:** 93–102.

Peltzer K, Promtussananon S. **Evaluation of Soul City school and mass media life skills education among junior secondary school learners in South Africa**. *Social Behavior Perspectives* 2003; **31:** 825–34.

Ramirez-Valles J, Kuhns L, Manjarrez D. **Tal Como Somos/Just As We Are: An Educational Film to Reduce Stigma towards Gay and Bisexual Men, Transgender Individuals & Persons Living with HIV/AIDS.** *Journal of Health Communications.* 2014 Apr; **19(4):** 478–492.

Rogers E, Vaughan P, Swalehe R, Rao N, Svenkerud P, Sood S. **Effects of an Entertainment-education Radio Soap Opera on Family Planning Behaviour in Tanzania**. *Studies in Family Planning* 1999; Vol. **30**, No. 3, pp. 193-211

Sakha M, Kazerooni P, Lari M, Sayadi M, Azar F, Motazedian N. **Effect of an educational intervention on knowledge, attitudes and preventive behaviours related to HIV and sexually transmitted infections in female sex workers in southern Iran: a quasi-experimental study.** *International Journal STD AIDS.* 2013 Sep; **24(9**):727-35

Shapiro D, Meekers D, Tambashe B. **Exposure to the ‘SIDA dans la Cite’ AIDS prevention television series in Cote d’Ivoire, sexual risk behaviour and condom use.** *AIDS Care* 2003; **15:** 303–14.

[Silvestre E](http://www.ncbi.nlm.nih.gov/pubmed/?term=Silvestre%20E%5BAuthor%5D&cauthor=true&cauthor_uid=26329265), [Weiner R](http://www.ncbi.nlm.nih.gov/pubmed/?term=Weiner%20R%5BAuthor%5D&cauthor=true&cauthor_uid=26329265), [Hutchinson P](http://www.ncbi.nlm.nih.gov/pubmed/?term=Hutchinson%20P%5BAuthor%5D&cauthor=true&cauthor_uid=26329265). **Behavior change communication and mobile populations: the evaluation of a cross-border HIV/AIDS communication strategy amongst migrants from Swaziland.** [*AIDS Care.*](http://www.ncbi.nlm.nih.gov/pubmed/26329265) 2015 Sep 2:1-7.

Smith R, Downs E, Witte K. **Drama Theory and Entertainment Education: Exploring the Effects of a Radio Drama on Behavioral Intentions to Limit HIV Transmission in Ethiopia.** *Communication Monographs* 2007; Vol. **74,** No. 2, pp. 133_153

Storey D, Boulay M, Karki Y, Heckert K, Karmacharya D. **Impact of the integrated radio communication project in Nepal, 1994-1997.** *Journal of Health Communication* 1999; **4** , 271-294.

Vaughan PW, Regis A, and St. Catherine E. (2000). **Effects of an entertainment-education radio soap opera on family planning and HIV prevention in St. Lucia.** *International Family Planning Perspectives*, 148-157.

Vernon R, Ojeda G, Murad R. **Incorporating AIDS prevention activities into family planning organization in Colombia.** *Stud Fam Plann* 1990; **21**: 335–43.

Whittier DK, Kennedy MG, St. Lawrence JS, Seeley S, and Beck V. (2005). **Embedding health messages into entertainment television: Effect on gay men's response to a syphilis outbreak.** *Journal of Health Communication*, **10(3),** 251-259.

Yoder P, Hornik R, Chirwa C. **Evaluating the program effects of a radio drama about AIDS in Zambia.** *Studies of Family Planning* 1996; **27:** 188–203.
